# Utilizing Big Data From Google Trends to Map Population Depression in the United States: Exploratory Infodemiology Study

**DOI:** 10.2196/35253

**Published:** 2022-03-31

**Authors:** Alex Wang, Robert McCarron, Daniel Azzam, Annamarie Stehli, Glen Xiong, Jeremy DeMartini

**Affiliations:** 1 Department of Psychiatry and Human Behavior University of California, Irvine Orange, CA United States; 2 Department of Psychiatry & Behavioral Sciences University of California, Davis Sacramento, CA United States

**Keywords:** depression, epidemiology, internet, google trends, big data, mental health

## Abstract

**Background:**

The epidemiology of mental health disorders has important theoretical and practical implications for health care service and planning. The recent increase in big data storage and subsequent development of analytical tools suggest that mining search databases may yield important trends on mental health, which can be used to support existing population health studies.

**Objective:**

This study aimed to map depression search intent in the United States based on internet-based mental health queries.

**Methods:**

Weekly data on mental health searches were extracted from Google Trends for an 11-year period (2010-2021) and separated by US state for the following terms: “feeling sad,” “depressed,” “depression,” “empty,” “insomnia,” “fatigue,” “guilty,” “feeling guilty,” and “suicide.” Multivariable regression models were created based on geographic and environmental factors and normalized to the following control terms: “sports,” “news,” “google,” “youtube,” “facebook,” and “netflix.” Heat maps of population depression were generated based on search intent.

**Results:**

Depression search intent grew 67% from January 2010 to March 2021. Depression search intent showed significant seasonal patterns with peak intensity during winter (adjusted *P*<.001) and early spring months (adjusted *P*<.001), relative to summer months. Geographic location correlated with depression search intent with states in the Northeast (adjusted *P*=.01) having higher search intent than states in the South.

**Conclusions:**

The trends extrapolated from Google Trends successfully correlate with known risk factors for depression, such as seasonality and increasing latitude. These findings suggest that Google Trends may be a valid novel epidemiological tool to map depression prevalence in the United States.

## Introduction

Over the past few decades, the amount of data stored, transferred, and analyzed has grown extensively, with the big data market reaching a value of US $139 billion in 2020 [1]. The term “big data” was coined in 2005 in reference to a large set of data that was essentially impossible to manage and process using traditional methods and tools [2]. As industries and companies have developed analytic tools targeted toward big data, information that was once inaccessible is now obtainable. One of the most important applications of big data in medicine is extrapolating trends and using them to support health care groups and organizations seeking to understand population health changes and predict the future.

Google Trends is a free online tool developed by Google LLC in 2008 that allows users from anywhere in the world to analyze big data [3]. It tracks search content across various countries and languages and compares relative search intent between 2 or more terms. The usefulness of Google Trends was demonstrated in 2009: Ginsberg et al [4] published a groundbreaking study predicting the spread of influenza earlier than the Centers for Disease Control and Prevention (CDC). Google Trends was subsequently utilized to predict the outbreaks of many viruses, including the West Nile virus, norovirus, varicella, influenza, and HIV [5-8]. More recently, Google Trends has been frequently used to study a variety of health care domains, including the COVID-19 pandemic [9].

Depression is the most common psychiatric disorder in the United States, with 18.5% of adults experiencing symptoms of depression in 2019 [10,11]. Since the start of the COVID-19 pandemic, the prevalence of depression symptoms has increased to 27.8%, affecting an estimated 91.2 million Americans [12]. Epidemiological data for depression have traditionally been collected through surveys. Major organizations such as the National Institute of Mental Health (NIMH), Anxiety & Depression Association of America (ADAA), and CDC provide only limited data specific to the time and population being studied from their surveys [13-15]. In response to the COVID-19 pandemic, the CDC and US Census Bureau collaborated to track mental health in the United States [16].

In this study, we provide estimates of depression search intent across the United States using big data from Google Trends. Our analysis fills the gap in current depression epidemiology, which is mainly derived from voluntary surveys, by extrapolating trends from big data across time and space. We provide an analysis of how internet search intent can be used to map population depression and how this can be compared in relation to depression risk factors. This model serves as a proof of concept that analyzing big data in association with environmental and geographic factors can be used as an epidemiological tool for psychiatric disease surveillance models. In terms of population health, analysis of Google Trends depression search intent represents a digital epidemiological tool that may one day be used for real-time surveillance of high-risk and underserved populations. The trends accessed through internet data may one day guide public policies, workforce supply decisions, and allocation of resources.

## Methods

### Google Trends

The following methodologies were designed based on published methods [17-19]. All search queries entered into Google’s search engine become anonymized and grouped based on both the general query topic and the specific keywords entered. Google Trends interprets the information and normalizes the data into an index between 0 and 100. The numbers represent the search interest relative to the highest point based on the given location and time frame within the query. A value of 100 represents highest search popularity for a term, and a value of 50 represents half the search popularity for a term [20].

To examine the US population’s interest in depression, we completed a series of search queries in Google Trends between January 1, 2010 and March 1, 2021. Data sets were downloaded for symptoms and terms listed by the American Psychiatric Association for major depressive disorder: “feeling sad,” “depressed,” “depression,” “empty,” “insomnia,” “fatigue,” “guilty,” “feeling guilty,” and “suicide” [21]. To account for random variance and overall increases in search queries, data sets were also downloaded across similar time periods for control terms based on previously published studies and popular internet search terms: “sports,” “news,” “google,” “youtube,” “facebook,” and “netflix” [18,19,22]. The values of depression search intent were summed and normalized relative to the control terms for the given region and time and are represented on a scale of 0 to 100 arbitrary units (AU).

Two separate data sets were extracted from Google Trends. The first data set represents the entire US public interest in depression over time with a data frequency of monthly averages from January 1, 2010 to March 1, 2021. The second data set represents public interest in depression on a statewide level collected as a single value per state averaged from January 1, 2010 to March 1, 2021.

### Environmental and Geographic Risk Factors

Given the known phenomenon of seasonal affective disorder, we obtained the annual temperature, humidity, and sunshine percentage from 1971 to 2000 from the National Climatic Center to assess for environmental and geographic risk factors of depression [23]. The sunshine percentage represents the percentage of time between sunrise and sunset that the sun reaches the earth’s surface. For the Air Quality Index (AQI), we obtained data from the 2010 to 2014 American Community Survey [24]. Values from the AQI were calculated for 4 major air pollutants regulated by the Clean Air Act [25]. Lastly, data for urban percentage were obtained from the 2010 US Census [26].

### Statistical Analysis

Multiple linear regression models were conducted to analyze the relationship between depression search queries and environmental factors and geographic factors. Confounding variables were identified using a correlation matrix and appropriately removed. The *P* values for each variable were adjusted according to the Bonferroni correction for multiple comparisons, with statistical significance determined at an adjusted *P*<.05. For predictive analysis, the multivariable regression models were constructed to generate quadratic forecasts to predict depression search intent and control search intent. The multiple regression models allowed us to account for confounding variables and prevent ecological fallacies according to previously published methods [27,28]. The values for normalized depression search intent were categorized into 4 regions according to the US Census Bureau: Northeast, Midwest, South, and West [29]. Geographic heat maps were generated in Microsoft Excel 2018 (Microsoft Corporation, Redmond, WA) to visualize the relationship between state temperature and state depression search intent.

## Results

### Multivariable Regression Model and Predictive Analysis in Relation to Time and Seasonality

**Figure 1:**
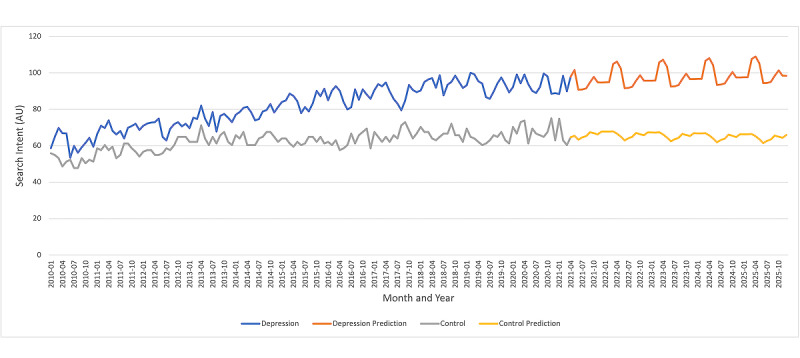
Time series plot of search intent for depression and control terms in the United States from 2010 to 2021 with predictive forecasts to 2025; demonstrates significant upward trend and seasonal pattern in depression search intent over time. AU: arbitrary unit.

The Google Trends data from January 2010 to March 2021 demonstrated an upward trend such that depression search intent grew 67% from 58.7 AU to 92.9 AU (n=135), while control search intent grew 24% to 67.1 AU (n=135). Based on the quadratic forecasts, depression search intent is predicted to increase an additional 7.4% to 99.8 AU in 2025 (95% CI 96.6 to 102.9 AU; n=135), while control search intent is predicted to increase 3.5% to 64.7 AU (95% CI 63.7 to 65.7 AU; n=135). A significant pattern of seasonality can be observed in Figure 1 with a peak in depression searches in the spring (March, April, May) and a trough in depression searches during the summer (June, July, August).

Table 1 presents the multivariable regression model using time and seasonality to predict depression search intent over time. The variables that were significant predictors of search intent were time (*r*=0.69, adjusted *P*<.001; n=135), time^2^ (*r*=0.91, adjusted *P*<.001; n=135), winter (*r*=0.03, adjusted *P*<.001; n=135), spring (*r*=0.12, adjusted *P*<.001; n=135), and fall (*r*=0.06, adjusted *P*<.001; n=135). Applying the regression model, there was a 0.5 AU (95% CI 0.42 to 0.57 AU; n=135) month-over month increase in depression search intent from 2010 to 2021. Depression search intent in the spring, fall, and winter were 7.0 AU (95% CI 5.3 to 8.7 AU; n=135), 4.6 AU (95% CI 2.9 to 6.4 AU; n=135), and 4.5 AU (95% CI 2.8 to 6.2 AU; n=135) higher than in summer, respectively.

**Table 1:** Multivariable regression model using time variables and season to predict seasonal depression search intent (*R*^2^=0.91).

Variables	Coefficients	Standard error	*t* statistic	*P* value	Adjusted *P* value^a^	*r*
Intercept	56.4	4.7	12.1	<.001	<.001	–^b^
Control	0.0	0.1	–0.4	.70	.99	0.69
Time	0.5	0.0	12.9	<.001	<.001	0.91
Time^2^	0.0	0.0	–6.8	<.001	<.001	0.83
Winter^c^	4.5	0.9	5.3	<.001	<.001	0.03
Spring^c^	7.0	0.9	8.2	<.001	<.001	0.12
Fall^c^	4.6	0.9	5.2	<.001	<.001	0.06

^a^Bonferroni correction for 4 independent analyses on the dependent variable (alpha=.05).

^b^Not applicable.

^c^Relative to summer.

### Multivariable Regression Model in Relation to Environmental and Geographic Risk Factors

**Figure 2:**
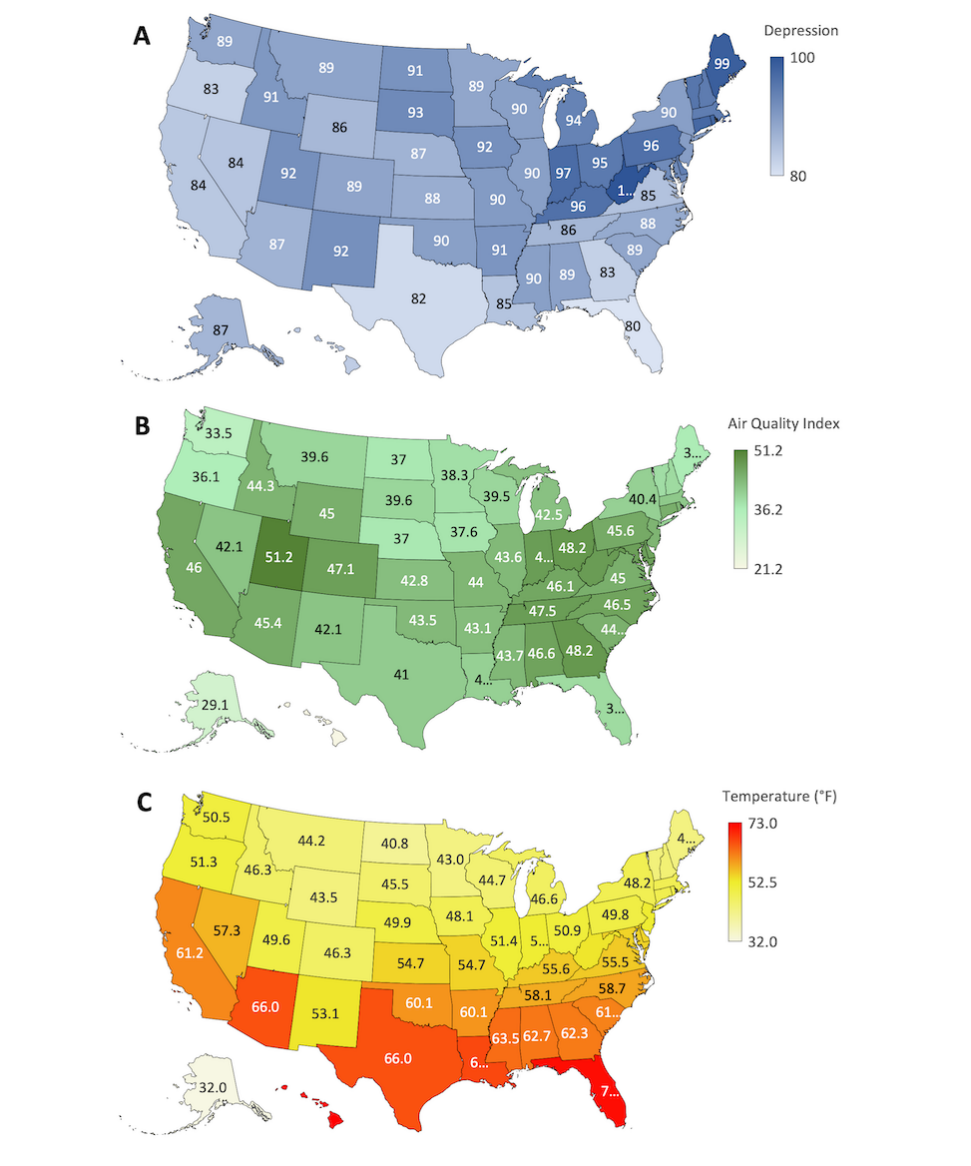
Geographic heat maps of the United States visualizing depression search intent on (A) Google Trends, (B) Air Quality Index, and (C) average annual temperature (° F) by state.

Table 2 presents the multivariable regression model of depression search intent based on state-specific environmental and geographic factors and has a predictive value of *R*^2^=0.57. In this model, variables that were significant predictors of depression search intent were AQI (*r*=0.30, adjusted *P*=.01; n=50) and the South (*r*=–0.2, adjusted *P*=.01; n=50). Applying the regression model, as AQI increased by 1, the depression search intent increased by 0.4 AU (95% CI 0.14 to 0.61 AU; n=50). Examining the depression search intent relative to US census regions, the South had a decrease of 6.3 AU (95% CI –10.2 to –2.3, adjusted *P*=.01; n=50) relative to the Northeast. Figure 2 visually demonstrates the regional differences such that states in the South such as Florida and Texas had lower depression search intent in comparison with states in the Northeast such as Maine and Pennsylvania. No relationships existed between depression search intent and temperature (*r*=–0.5, adjusted *P*=.99; n=50), humidity (*r*=0.2, adjusted *P*=.99; n=50), urban percentage (*r*=0.3, adjusted *P*=.06; n=50), or sunshine percentage (*r*=–0.5, adjusted *P*=.99; n=50).

**Table 2:** Multivariable regression model of depression search intent in relation to environmental and geographic risk factors (*R*^2^=0.57).

Variables^a^	Coefficients	Standard error	*t* statistic	*P* value	Adjusted *P* value^b^	*r*
Intercept	94.9	12.2	7.8	<.001	<.001	-^c^
Temperature	0.0	0.1	–0.3	.74	.99	–0.5
Humidity	0.0	0.1	0.1	.89	.99	0.2
Air Quality Index	0.4	0.1	3.2	.002	.01	0.3
Urban %	–0.1	0.0	–2.7	.01	.06	0.3
Sunshine %	–9.0	13.1	–0.7	.50	.99	–0.5
South^d^	–6.3	1.9	–3.2	.002	.01	–0.2
West	–4.4	1.8	–2.5	.02	.11	–0.3
Midwest	–3.8	1.6	–2.4	.02	.12	0.1

^a^Multivariable regression model using environmental and geographic risk variables to predict depression search intent. Environmental and geographic data sets were collected as an average from 1971 to 2000 and 2008 to 2019, respectively (n=50). This model predicts depression search intent for each state based on the state's average annual temperature, humidity, air quality, urban %, sunshine %, and US census region.

^b^Bonferroni correction for 6 independent analyses on the dependent variable (alpha=.05).

^c^Not applicable.

^d^Relative to the Northeast.

## Discussion

### Principal Findings

To our knowledge, this is the first study to geographically map depression search intent across the United States in relation to environmental and geographic risk factors by using statistical analysis of big data through Google Trends. Traditionally, prevalence data for mental health and depression have been collected through surveys that require an intensive amount of time and resources to conduct [12-14,30,31]. These surveys are limited not only by human and monetary resources but also by participants’ willingness to be included in research. According to the National Survey on Drug Use and Health (SAMHSA), 32.9% of the selected sample did not complete the interview because of refusal to participate, absence from their home, language barriers, or other reasons such as physical or mental incompetence [32]. Response bias is a known recurring issue with epidemiological surveys and has been difficult to overcome as patients with severe mental health and the homeless population are continuously marginalized by society [33].

The solution to this problem may be utilization of big data found through the internet. In 2020, roughly 86% of the total US population had access to the internet [34]. A US study in greater Los Angeles that examined digital technology use in homeless populations discovered that 94% owned a cell phone [35]. Currently where digital technology is a requirement for survival, internet data can be used to track populations from all over the world over any period. The use of real-time monitoring of internet data to track trends and diseases overcomes the issues of resources, time, and physical location. Analyzing big data through Google Trends is free to researchers and provides information and predictive insight that may one day surpass national or local surveillance systems.

### Comparison With Prior Work

The validity of using big data for epidemiology was demonstrated during the influenza outbreak of 2009. At the time, Google Trends was an experimental tool used by researchers for real-time monitoring of influenza outbreaks [36]. By analyzing health care info-seeking behavior on the Google search engine, Google Trends was able to detect regional outbreaks of influenza 7-10 days before the CDC. Google Trends has been successfully used to track viral outbreaks and is currently being used to monitor COVID-19 outbreaks across the world [4-9,37,38].

Depression is a major public health concern and one of the most prevalent mental health illnesses in the United States [10,39]. In 2010, the estimated annual economic consequence of depression was upwards of US $200 billion [40,41]. Considering depression also leads to diminished productivity, poor quality of life, and negative psychological impacts on well-being, the true costs of depression on society are much higher [42]. Worsening mental health and an increasing prevalence of depression, especially during the COVID-19 pandemic, signify the increasing importance of monitoring and treating patients with depression. Based on our analysis, Google search intent for depression in the United States has grown by 67% from 2010 to 2021 and is projected to grow another 7.4% by 2025. This increase reflects the epidemiological trends reported by US national surveys, with an increase in depression prevalence by 61% from 2008 to 2018 (6.6% to 10.4%) [43,44]. This corroborates the concept that, as depression prevalence in the United States continues to grow, so does the information-seeking behavior on Google Trends. Furthermore, depression search intent in the United States demonstrated a significant seasonal pattern, such that depression search intent was lowest in the summer. Relative to the summer, the fall, winter, and spring seasons had an increase in depression search intent by 4.6 AU, 4.5 AU, and 7.0 AU, respectively. This increase in depression search intent reflects the seasonal pattern of seasonal affective disorder (SAD) which has been shown to have higher prevalence in the fall and winter seasons and a decrease in the summer [45,46]. Although SAD has been shown to begin remission in the spring, the increase in depression search intent in the spring may reflect population interest in depression in the early stages of a patient’s recovery.

In relation to environmental and geographic risk factors, the state’s air quality and geographic location had significant predictive values for depression search intent. States that had a 1-unit higher AQI had an increase in depression search intent by 0.4 AU In other words, states with worse air pollution had higher levels of depression search intent than states with cleaner air. These results reflect previously published findings that air pollution is linked to depression [47-49]. Our results comparing the 4 US census regions demonstrated that the South had less search intent, by 6.3 AU, relative to the Northeast. The West and Midwest also demonstrated decreased levels of depression search intent, by 4.4 AU and 3.8 AU, respectively, though their adjusted *P* values were insignificant. These results reflect the findings that depression is correlated with latitude, with regions further from the equator having a higher prevalence of depression [42,50]. Although the season and location of a state cannot fully predict the depression search intent at a given time, the trends extrapolated from Google Trends have demonstrated their validity in relation to known risks of depression.

Although mining for epidemiological trends within big data is a fascinating prospect, it should not be assumed to replace the work of national and public health organizations. Instead, researchers should consider comparing their results with big data and using big data to support their findings. Our study has demonstrated that depression search intent increased over time following a seasonal pattern and was higher in states with higher air pollution and states with northern latitudes. This supports the trends found in US epidemiological surveys on mental health and supports published results of known risk factors for depression.

Future studies should build upon the results demonstrated here by examining other risk factors for depression such as socioeconomic, demographic, or lifestyle variables. More specifically, whether age, income, marital status, race/ethnicity, or gender are predictive variables of depression search intent, both on national and state levels. Considering the COVID-19 pandemic, future studies should analyze the data based on advanced time series modeling to analyze the effects of the pandemic on mental health. In the future, public organizations such as the CDC or regional hospitals may be able to monitor depression prevalence in real time based on the search intent of their communities through publicly available internet data. The clinical applications of big data in the medical field are limitless and will continue to become more useful as technology software improves.

### Limitations

Several limitations are present in our study. First, interpreting the trends extrapolated from Google Trends is challenging without supporting clinical information normally collected by traditional surveys such as medical comorbidities or symptom severity. Second, the data in Google Trends may be influenced by various factors such as trending television shows or bots. For example, in 2017, the internet search intent for suicide queries increased by 19% over a 19-day span after the release of popular Netflix series, *13 Reasons Why*, which elevated suicide awareness [51]. Third, our data may overrepresent people that search terms in English as Google Trends does not combine search intent of the same word in another language. Fourth, the geographic and environmental data sets were consolidated into a single data point for each state regardless of varying climates and heterogenous landscapes. Lastly, patients with severe or debilitating depression may not have the capacity to search for depression or have the access if they are hospitalized. These limitations illustrate that overreliance on big data, much like on epidemiological studies, may inadvertently exclude certain populations.

### Conclusions

Our study is the first to demonstrate that big data in Google Trends can be successfully utilized as a novel epidemiological tool to geographically map out population depression in the United States. This method of mapping allows for easier visualization of areas with higher depression search intent, which were mostly states with higher air pollution and those further from the equator. The interest in depression has grown tremendously in the past decade, with an upward trend that follows a seasonal variation pattern similarly seen in SAD. AQI and geographic location were stronger predictors of depression search intent than temperature, humidity, urban percentage, or sunshine percentage. Further investigation is needed to determine whether the factors significant in our study hold true to depression trends across the world. From a clinical perspective, narrowing the scope of depression search intent to specific cities or high-risk populations should be the next goal of researchers.

## Abbreviations

ADAA: Anxiety & Depression Association of America
AQI: Air Quality Index
AU: arbitrary units
CDC: Centers for Disease Control and Prevention
NIMH: National Institute of Mental Health
SAD: seasonal affective disorder
SAMHSA: National Survey on Drug Use and Health
